# Supplementary figures and images for: The conserved centrosomin motif, γTuNA, forms a dimer that directly activates microtubule nucleation by the γ-tubulin ring complex (γTuRC)
Source: eLife. 2022 Dec 14;11:e80053. doi: 10.7554/eLife.80053 (PMC9859039; doi:10.7554/eLife.80053)

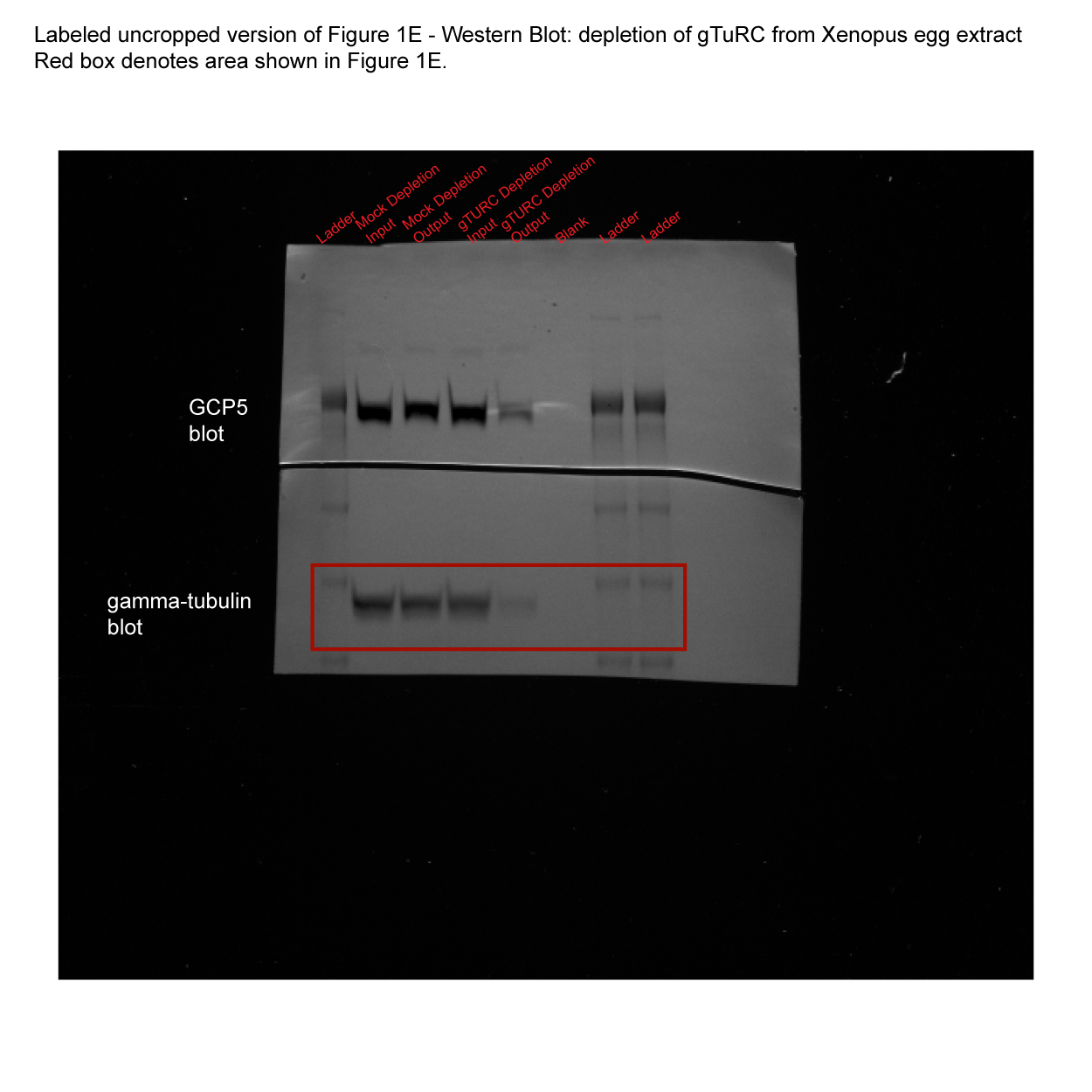

Supplement: Figure 1—source data 2. [file elife-80053-fig1-data2.zip › Figure 1 - Source Data 2/Labeled_uncropped_Fig1E-01.tif]

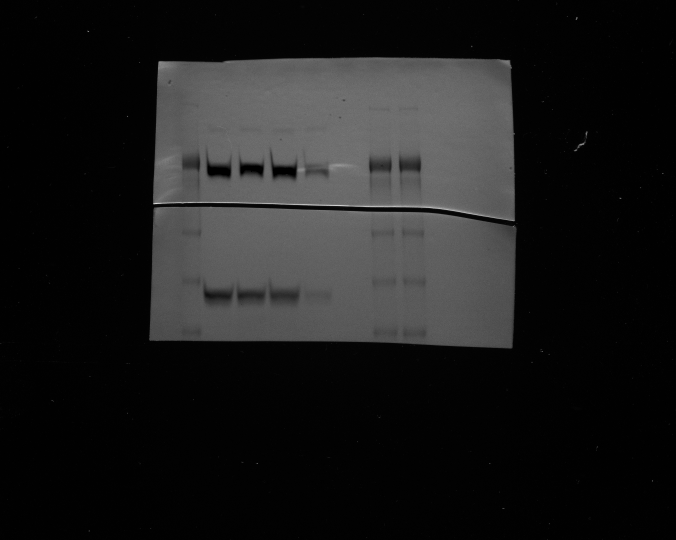

Supplement: Figure 1—source data 2. [file elife-80053-fig1-data2.zip › Figure 1 - Source Data 2/Raw blots/Figure1E_gTuRCdepletionExtract_Raw_3sec.tif]

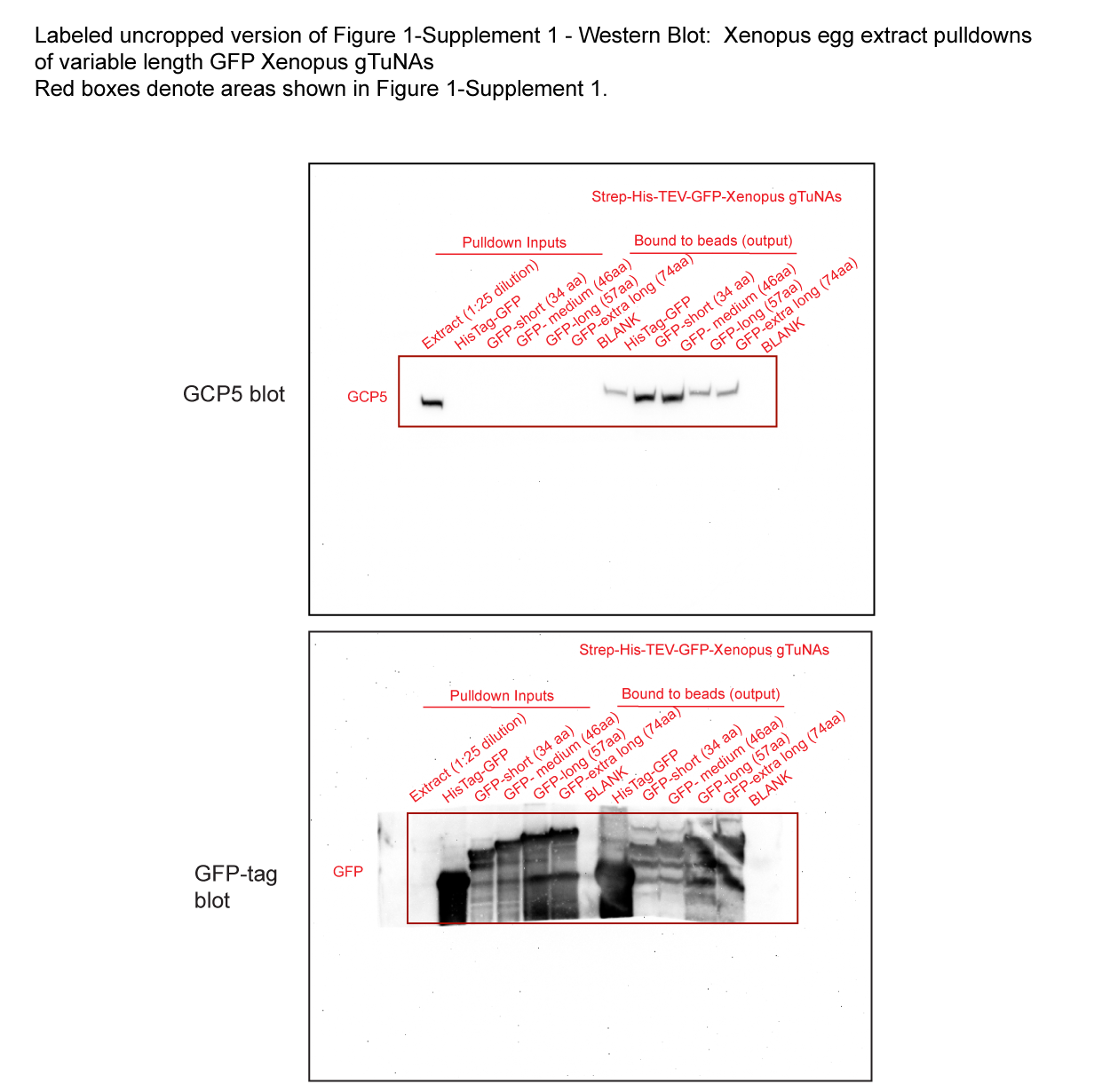

Supplement: Figure 1—figure supplement 1—source data 1. [file elife-80053-fig1-figsupp1-data1.zip › Figure 1 - Supplement 1 - Source Data 1/Labeled_uncropped_Fig1Suppl-1-01.tif]

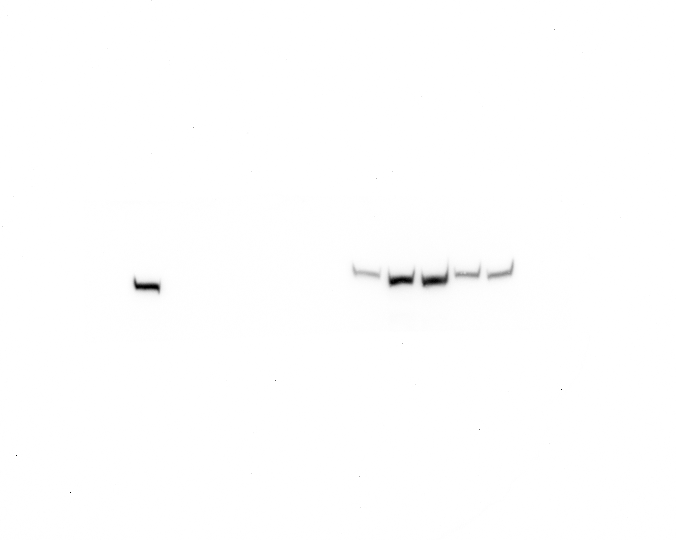

Supplement: Figure 1—figure supplement 1—source data 1. [file elife-80053-fig1-figsupp1-data1.zip › Figure 1 - Supplement 1 - Source Data 1/Raw blots/Figure1-Suppl1_chemiGCP5.tif]

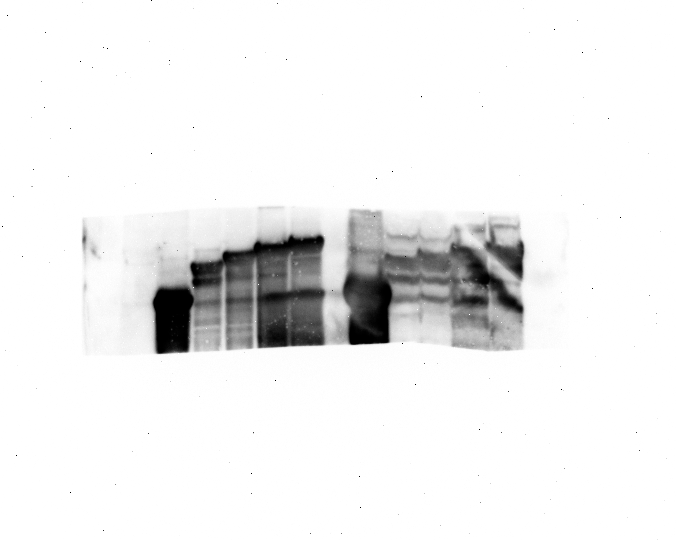

Supplement: Figure 1—figure supplement 1—source data 1. [file elife-80053-fig1-figsupp1-data1.zip › Figure 1 - Supplement 1 - Source Data 1/Raw blots/Figure1-Suppl1_chemionlyGFP.tif]

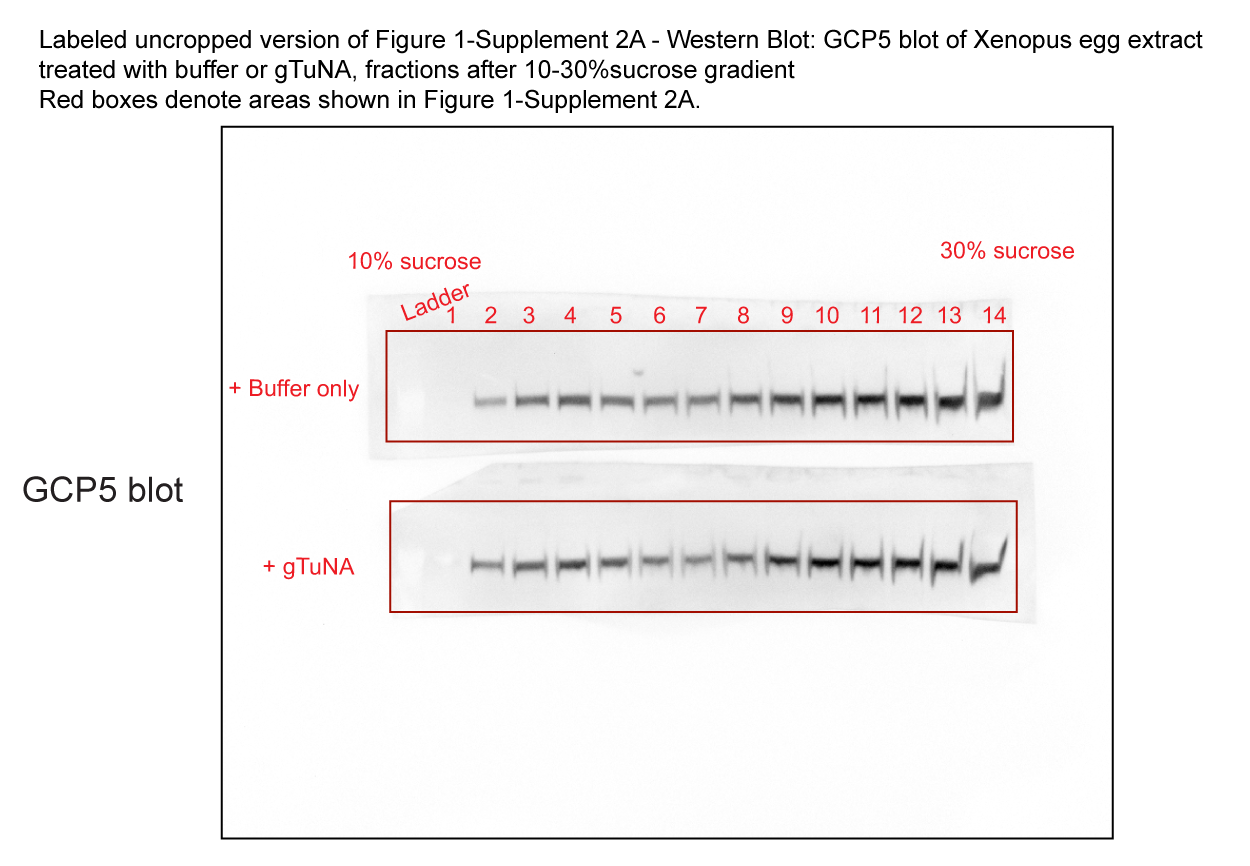

Supplement: Figure 1—figure supplement 2—source data 1. [file elife-80053-fig1-figsupp2-data1.zip › Figure 1 - Supplement 2 - Source Data 1/Figure1-Suppl2A/Labeled_uncropped_Fig1Suppl-2A-01.tif]

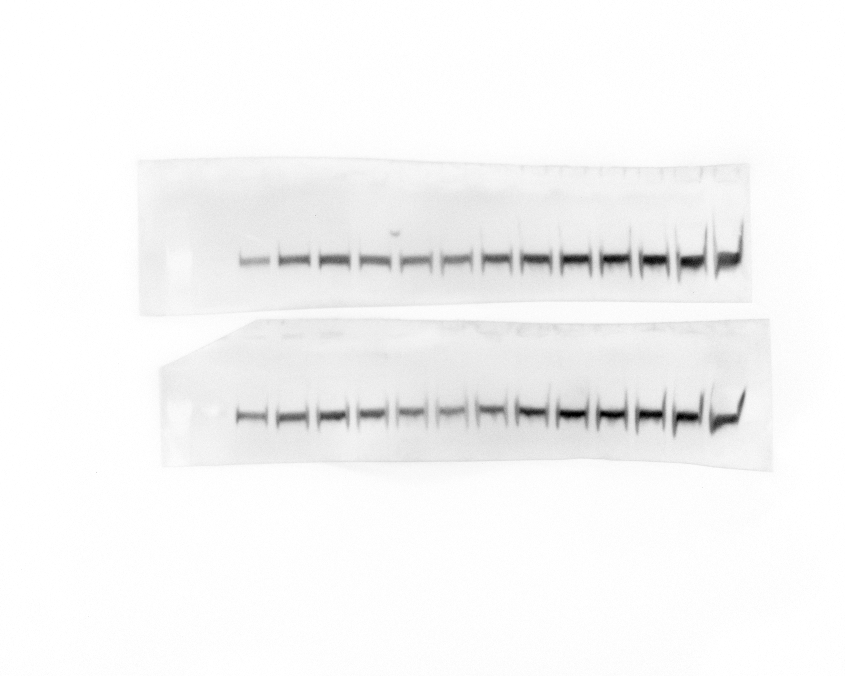

Supplement: Figure 1—figure supplement 2—source data 1. [file elife-80053-fig1-figsupp2-data1.zip › Figure 1 - Supplement 2 - Source Data 1/Figure1-Suppl2A/Raw blot/Figure1-Suppl2-A_GCP5.tif]

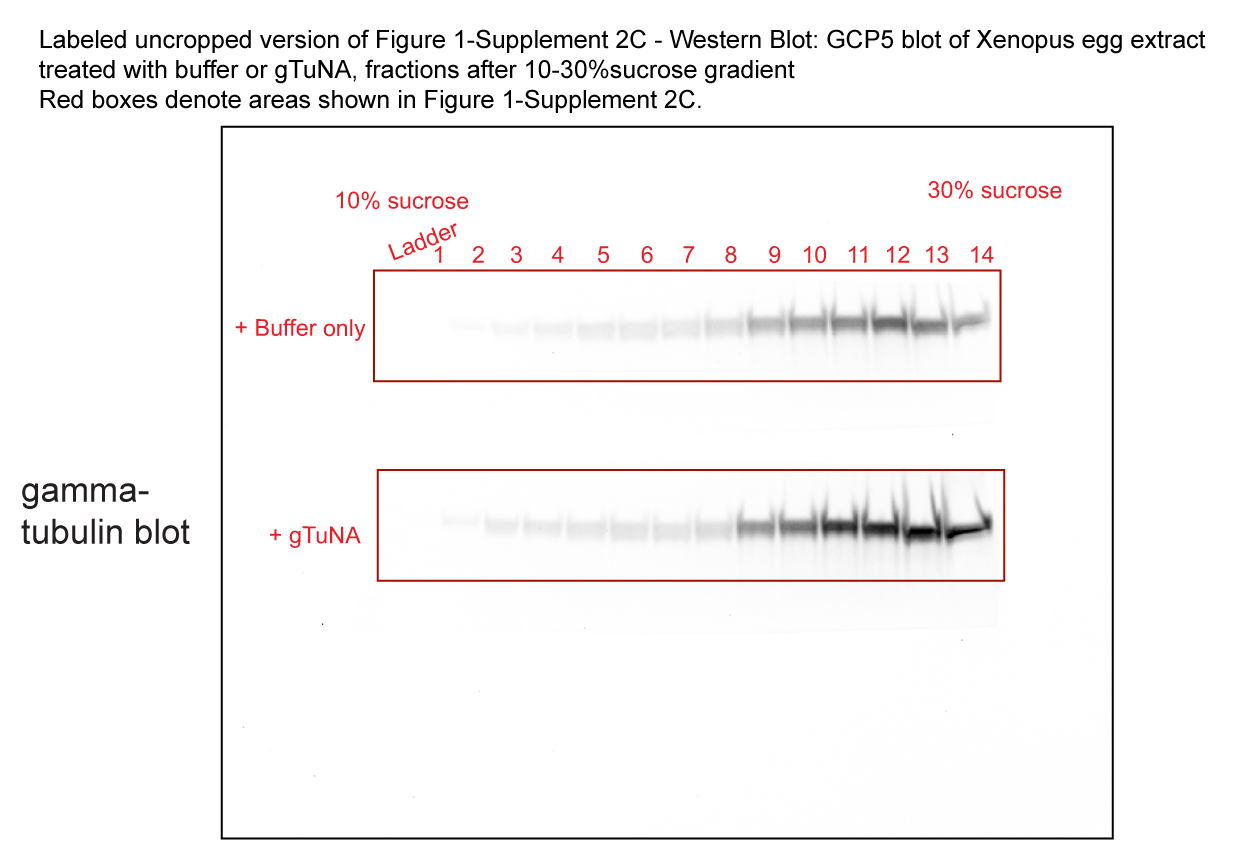

Supplement: Figure 1—figure supplement 2—source data 1. [file elife-80053-fig1-figsupp2-data1.zip › Figure 1 - Supplement 2 - Source Data 1/Figure1-Suppl2C/Labeled_uncropped_Fig1Suppl-2C-01.tif]

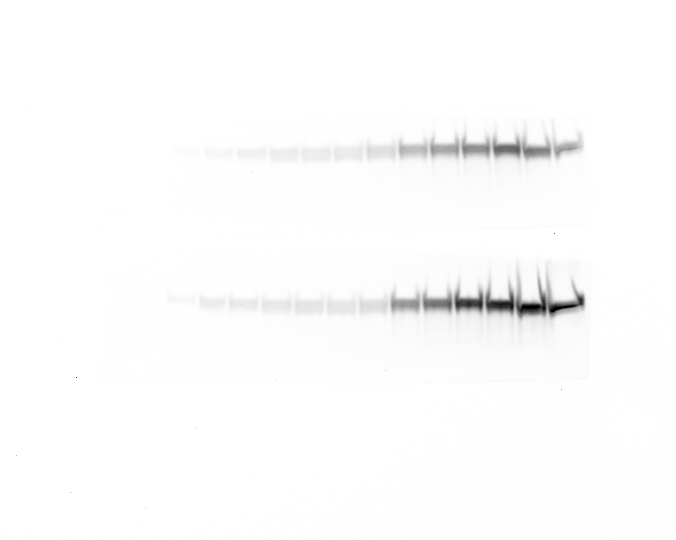

Supplement: Figure 1—figure supplement 2—source data 1. [file elife-80053-fig1-figsupp2-data1.zip › Figure 1 - Supplement 2 - Source Data 1/Figure1-Suppl2C/Raw blot/Figure1-Suppl2-C_gtub.tif]

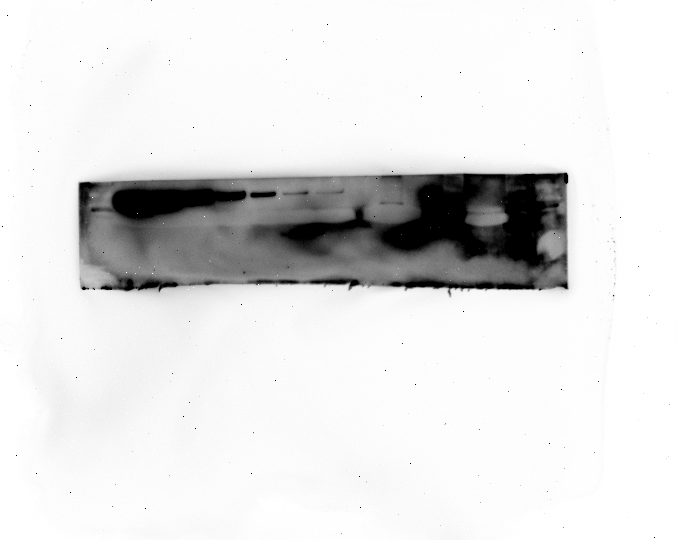

Supplement: Figure 2—figure supplement 2—source data 1. [file elife-80053-fig2-figsupp2-data1.zip › Figure 2 - Supplement 2 - Source Data 1/Fig2-Suppl2-C/Raw blots/Figure2-Suppl2-C_AU1blot_7min.tif]

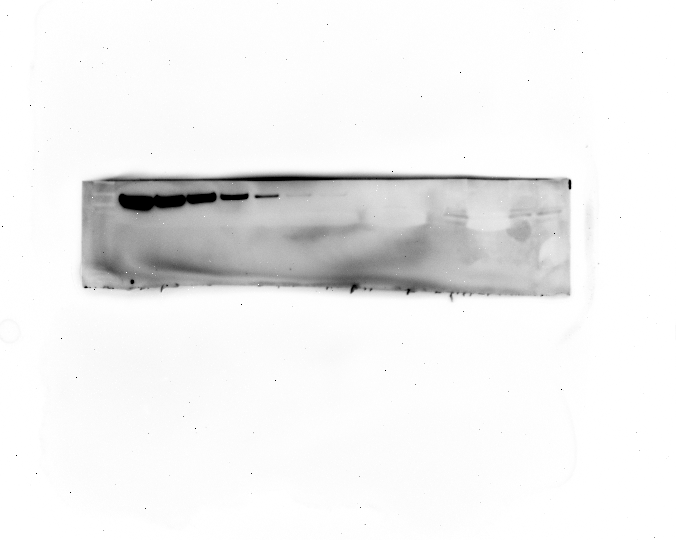

Supplement: Figure 2—figure supplement 2—source data 1. [file elife-80053-fig2-figsupp2-data1.zip › Figure 2 - Supplement 2 - Source Data 1/Fig2-Suppl2-C/Raw blots/Figure2-Suppl2-C_AU1blot_5min.tif]

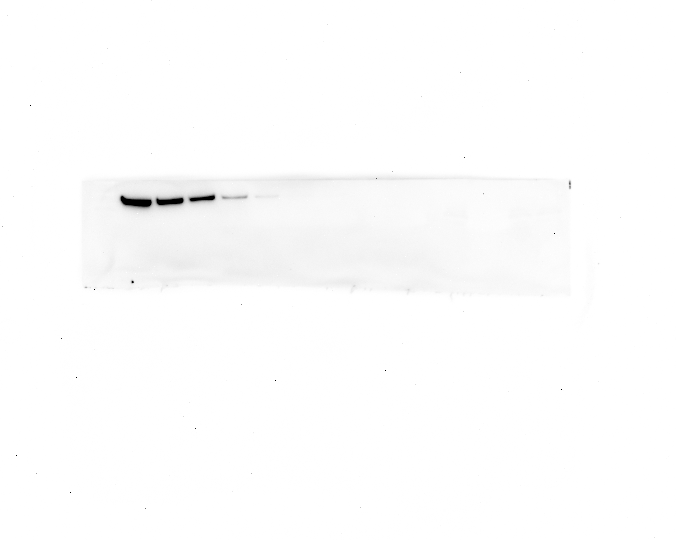

Supplement: Figure 2—figure supplement 2—source data 1. [file elife-80053-fig2-figsupp2-data1.zip › Figure 2 - Supplement 2 - Source Data 1/Fig2-Suppl2-C/Raw blots/Figure2-Suppl2-C_AU1blot_1min.tif]

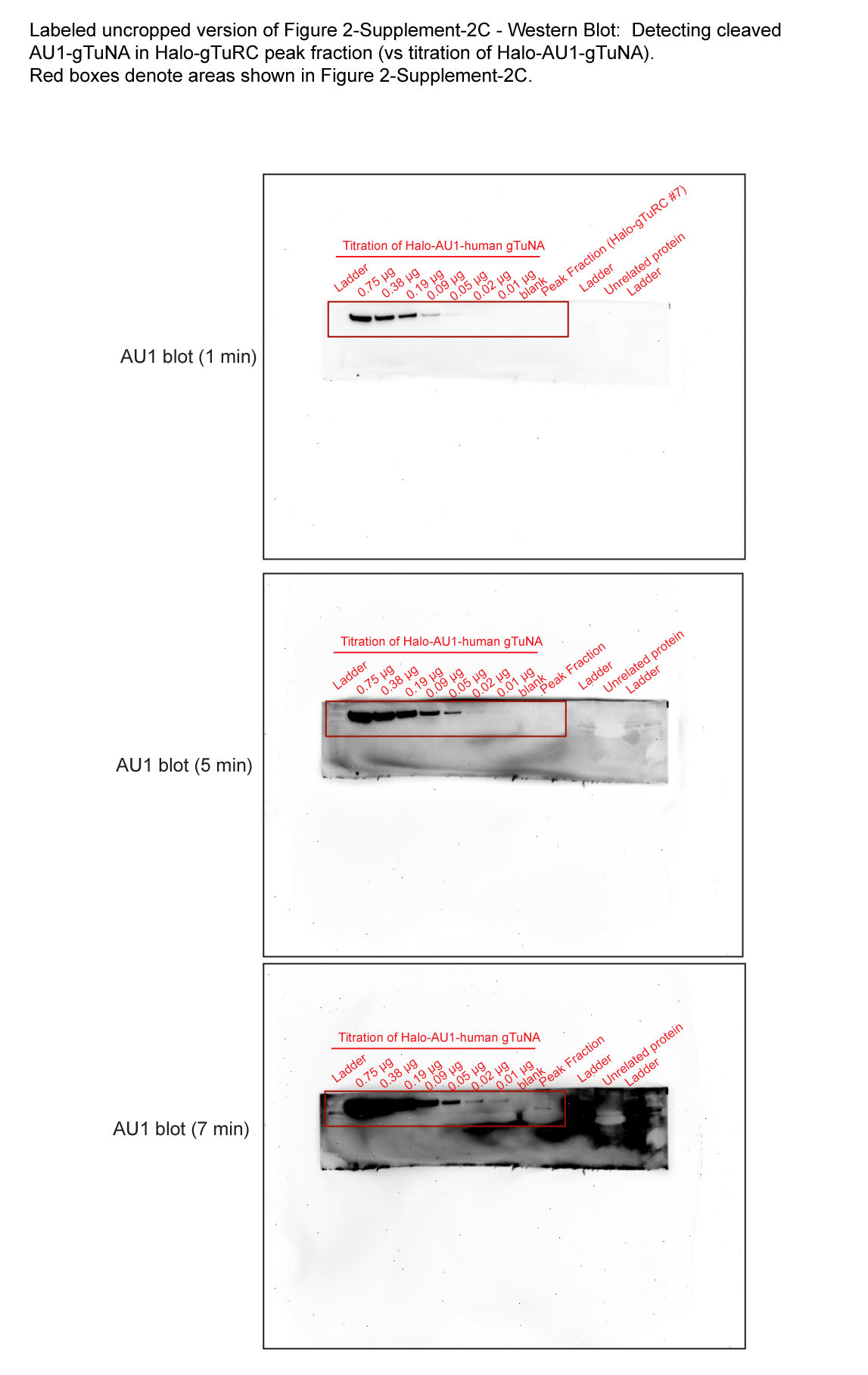

Supplement: Figure 2—figure supplement 2—source data 1. [file elife-80053-fig2-figsupp2-data1.zip › Figure 2 - Supplement 2 - Source Data 1/Fig2-Suppl2-C/Labeled_uncropped_Fig2-Suppl2-C-01.tif]

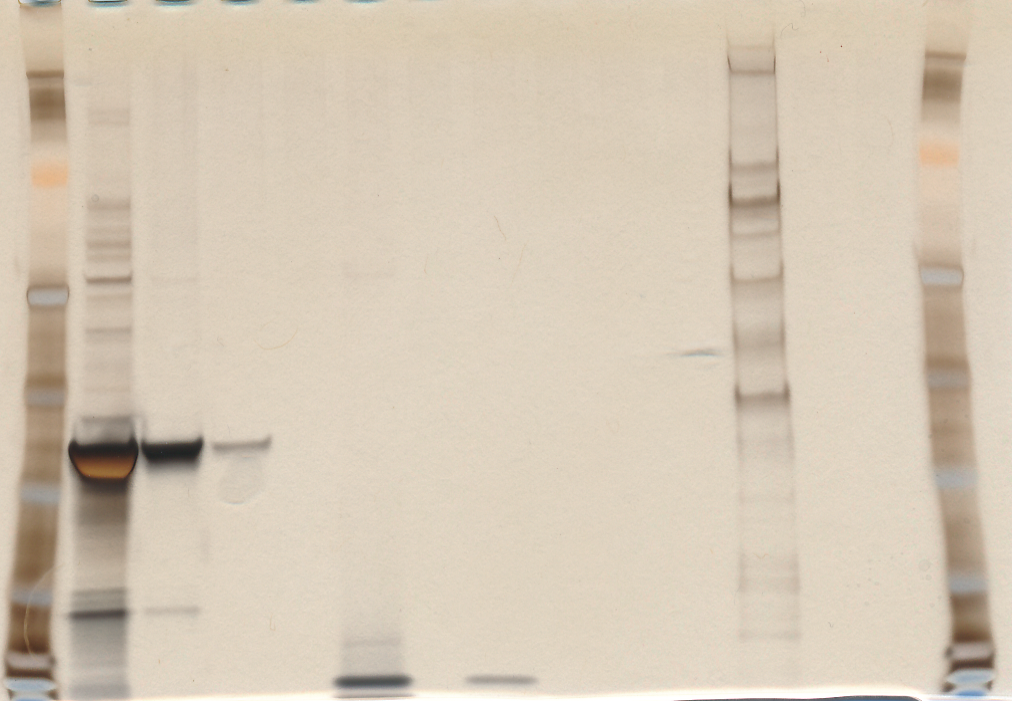

Supplement: Figure 2—figure supplement 2—source data 1. [file elife-80053-fig2-figsupp2-data1.zip › Figure 2 - Supplement 2 - Source Data 1/Fig2-Suppl2-B/Raw gel/Figure2-Suppl2-B_HalogTuRCFraction7_silverstaingel.tif]

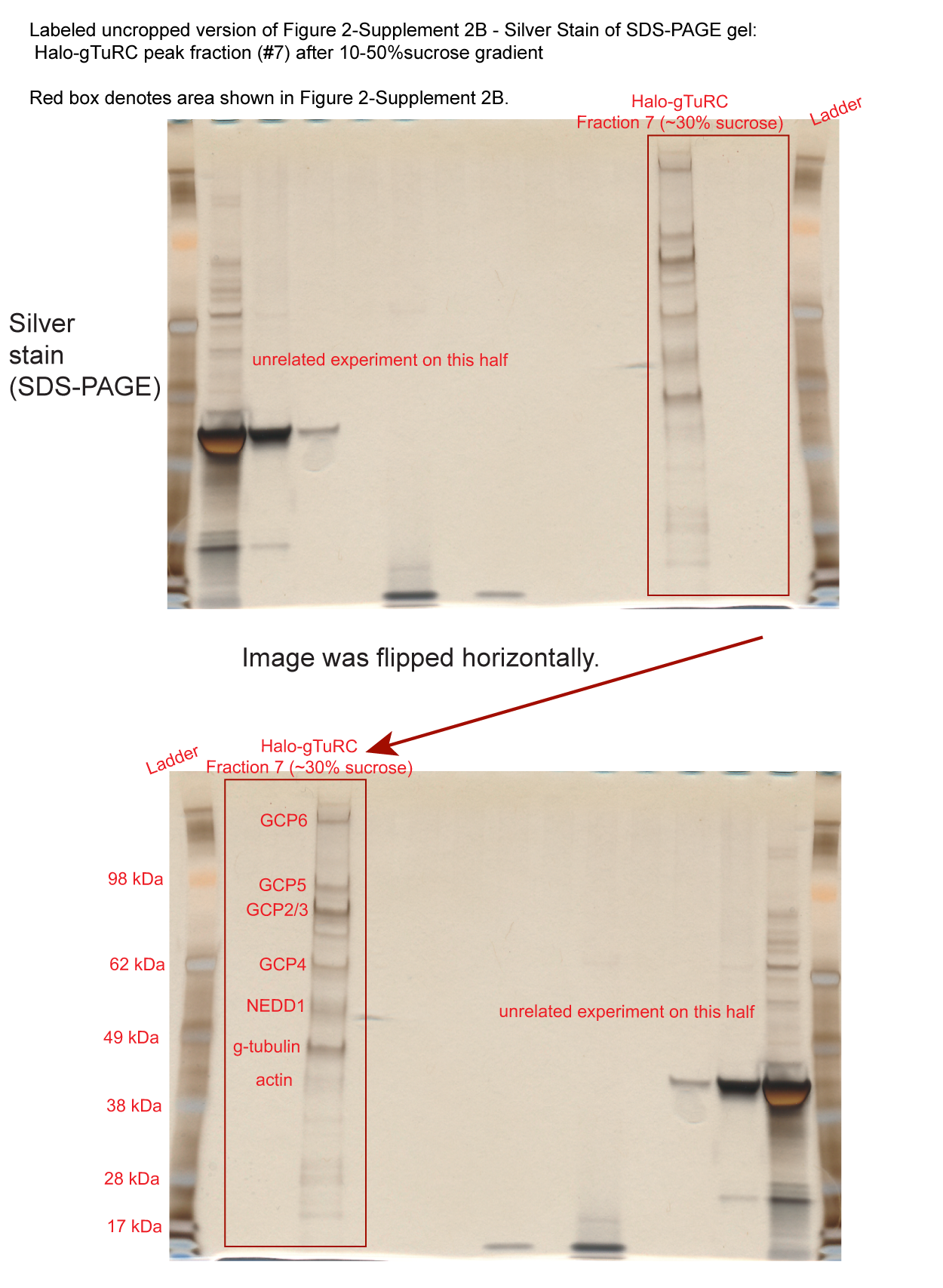

Supplement: Figure 2—figure supplement 2—source data 1. [file elife-80053-fig2-figsupp2-data1.zip › Figure 2 - Supplement 2 - Source Data 1/Fig2-Suppl2-B/Labeled_uncropped_Fig2Suppl-2B-01.tif]

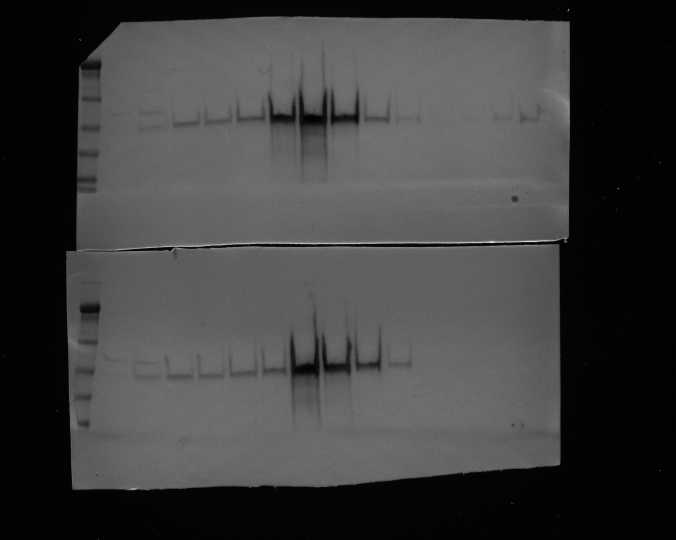

Supplement: Figure 2—figure supplement 2—source data 1. [file elife-80053-fig2-figsupp2-data1.zip › Figure 2 - Supplement 2 - Source Data 1/Fig2-Suppl2-A/Raw blots/Figure2-Supplement2-A_sucrosegradHalogTuRC.tif]

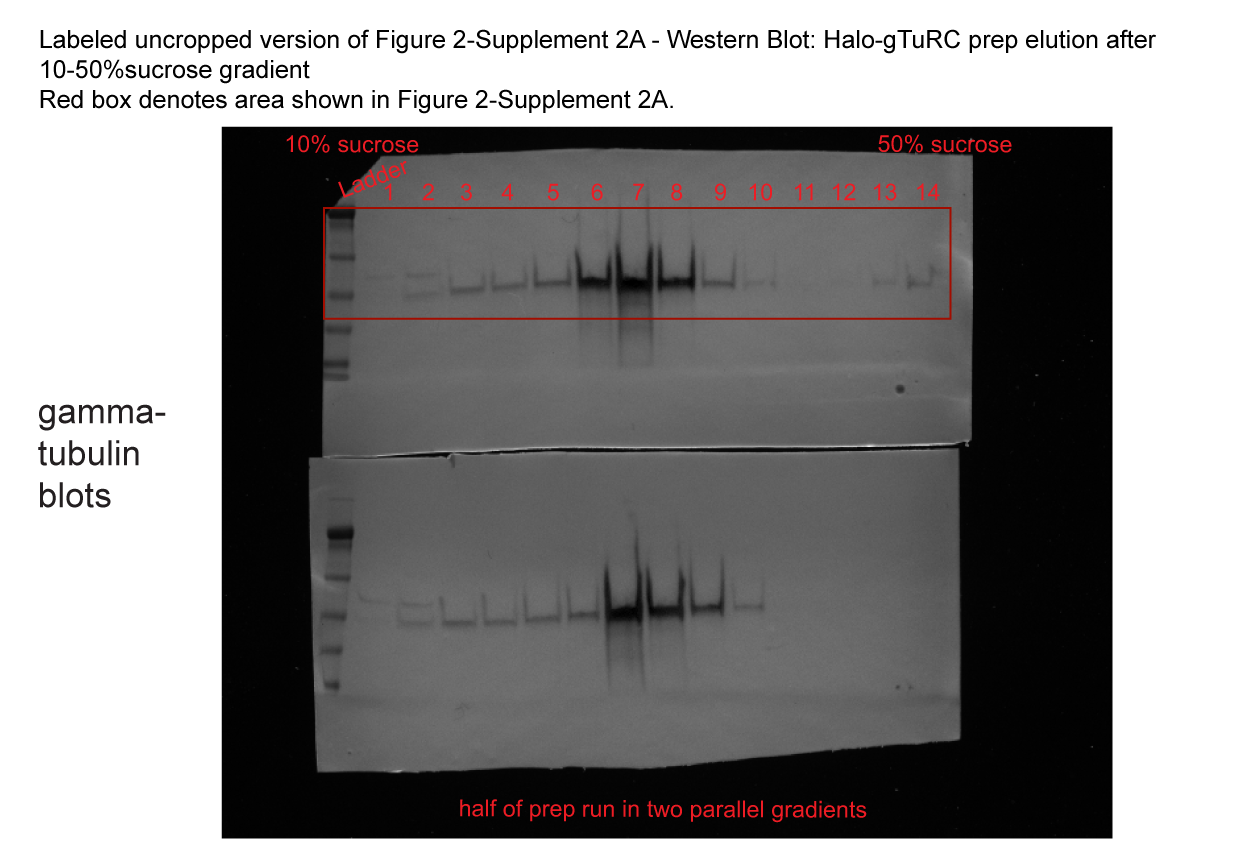

Supplement: Figure 2—figure supplement 2—source data 1. [file elife-80053-fig2-figsupp2-data1.zip › Figure 2 - Supplement 2 - Source Data 1/Fig2-Suppl2-A/Labeled_uncropped_Fig2Suppl-2A-01.tif]

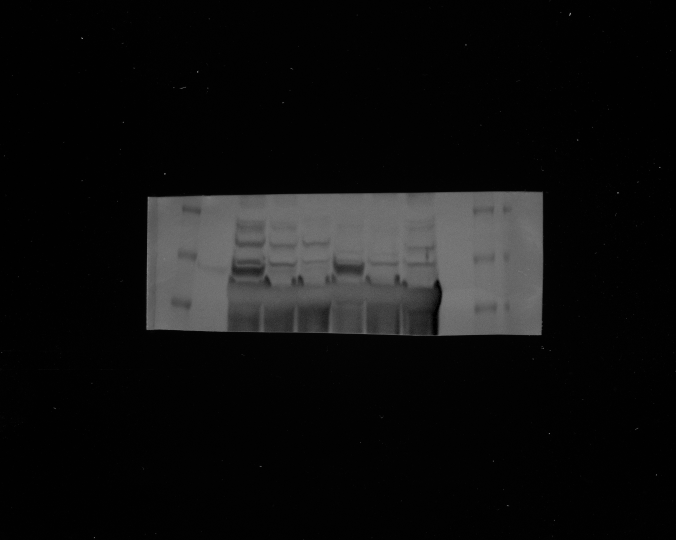

Supplement: Figure 3—source data 2. [file elife-80053-fig3-data2.zip › Figure 3 - Source Data 2/Figure3D/Raw blots/Figure3-D_strep.tif]

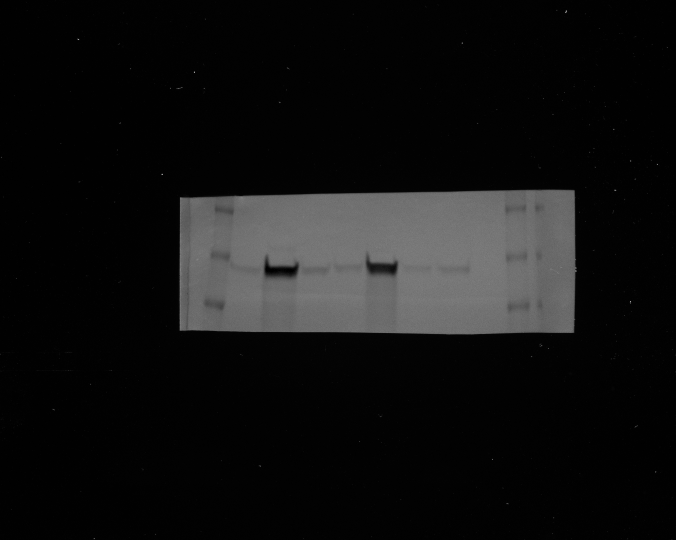

Supplement: Figure 3—source data 2. [file elife-80053-fig3-data2.zip › Figure 3 - Source Data 2/Figure3D/Raw blots/Figure3-D_gamma-Tub.tif]

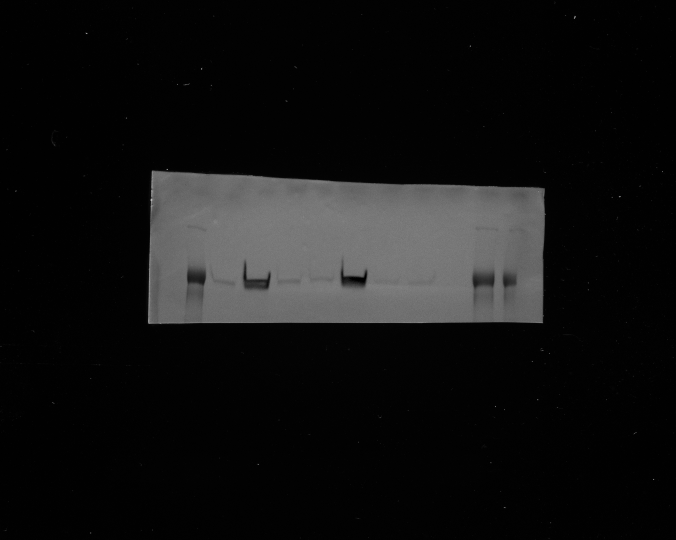

Supplement: Figure 3—source data 2. [file elife-80053-fig3-data2.zip › Figure 3 - Source Data 2/Figure3D/Raw blots/Figure3-D_GCP5.tif]

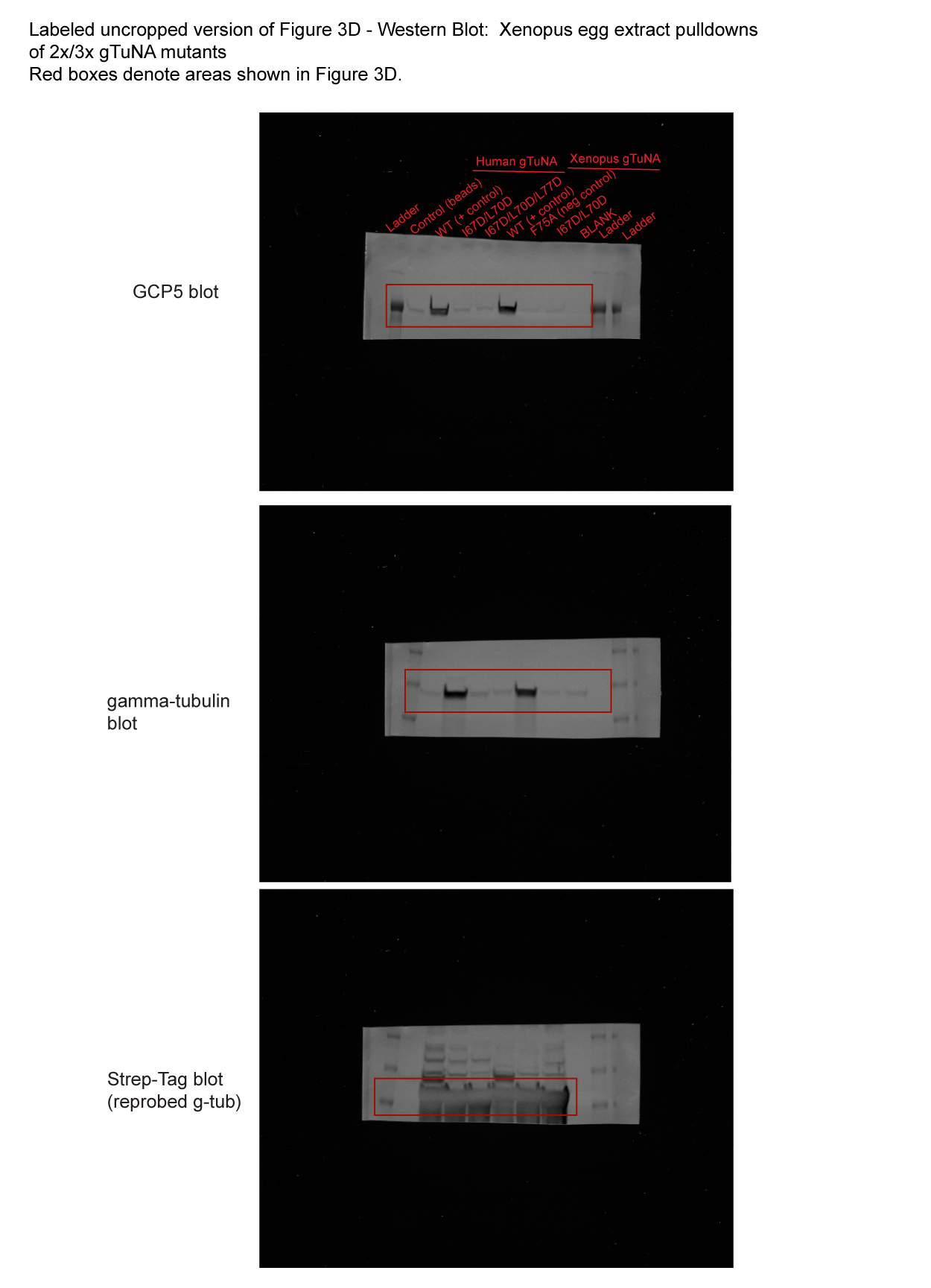

Supplement: Figure 3—source data 2. [file elife-80053-fig3-data2.zip › Figure 3 - Source Data 2/Figure3D/Labeled_uncropped_Fig3D-01.tif]

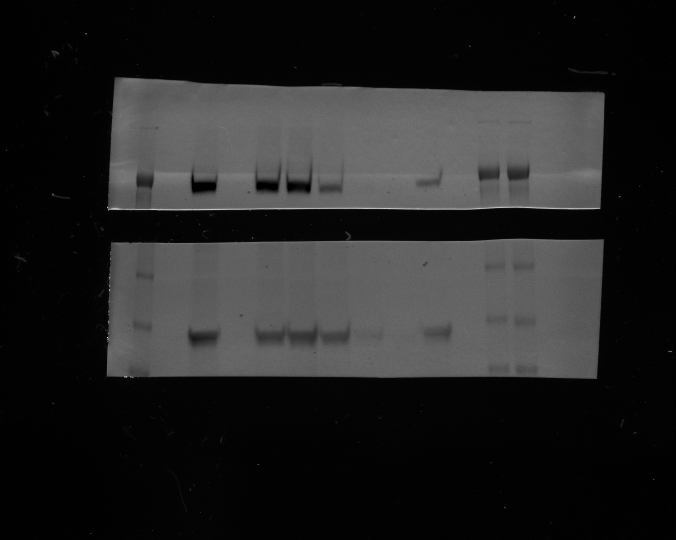

Supplement: Figure 3—source data 2. [file elife-80053-fig3-data2.zip › Figure 3 - Source Data 2/Figure3E/Raw blots/Figure3E_gcp5_gtub_500msec.tif]

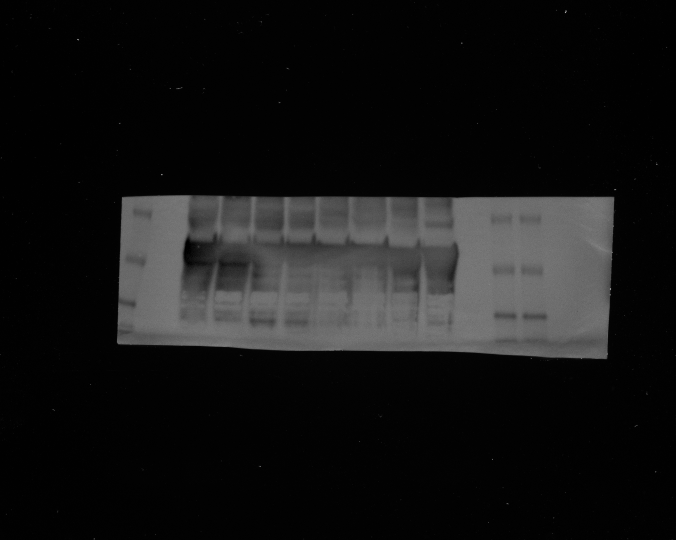

Supplement: Figure 3—source data 2. [file elife-80053-fig3-data2.zip › Figure 3 - Source Data 2/Figure3E/Raw blots/Figure3E_strep_200msec.tif]

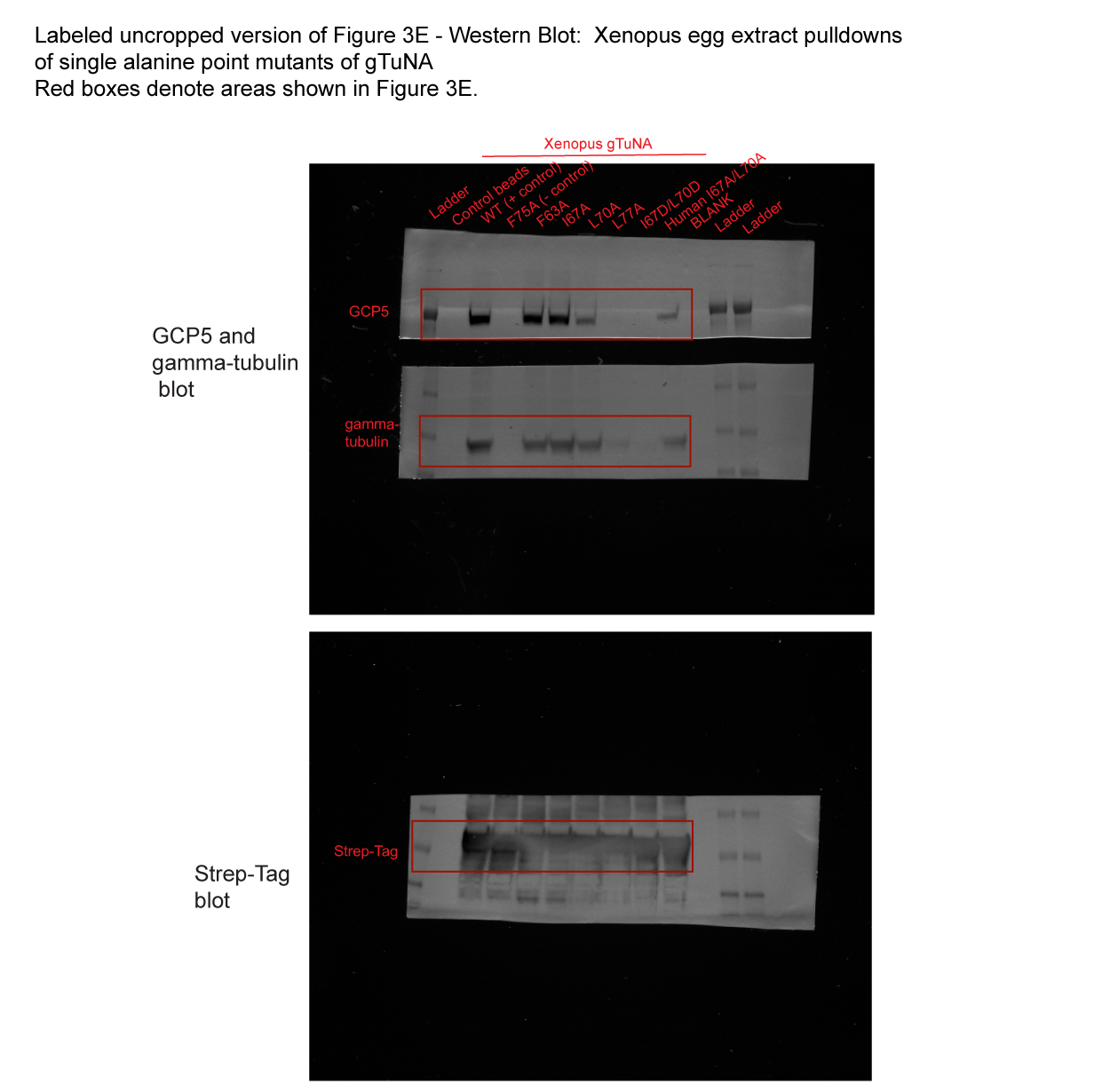

Supplement: Figure 3—source data 2. [file elife-80053-fig3-data2.zip › Figure 3 - Source Data 2/Figure3E/Labeled_uncropped_Fig3E-01.tif]

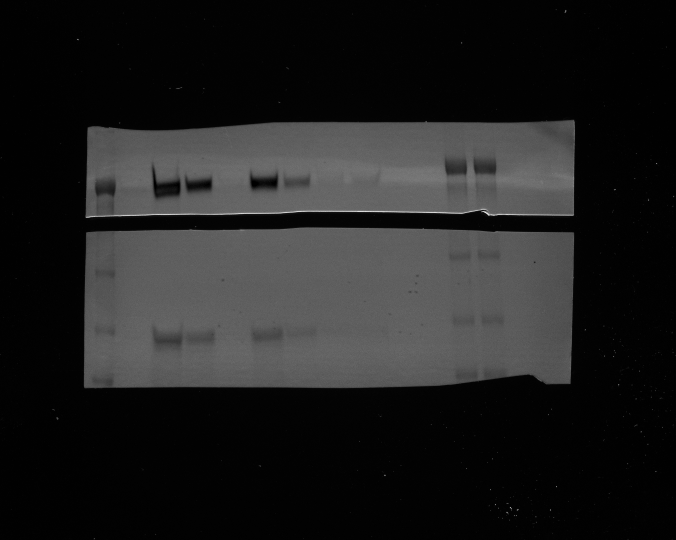

Supplement: Figure 3—figure supplement 1—source data 1. [file elife-80053-fig3-figsupp1-data1.zip › Figure 3 - Supplement 1- Source Data 1/Figure3-Suppl-B/Raw blots/Figure3-Suppl1-B_GCP5andgTUB_sourceOfGCP5band_300msec_TOP.tif]

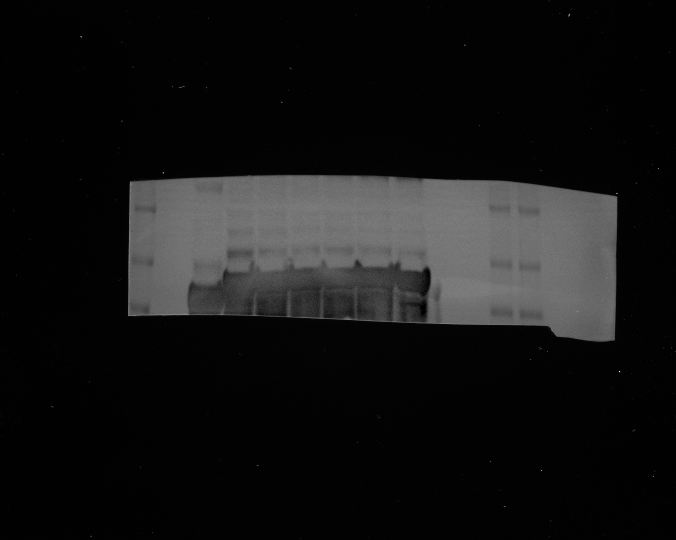

Supplement: Figure 3—figure supplement 1—source data 1. [file elife-80053-fig3-figsupp1-data1.zip › Figure 3 - Supplement 1- Source Data 1/Figure3-Suppl-B/Raw blots/Figure3-Suppl1-B_Strepblot_100msec_BOTTOM.tif]

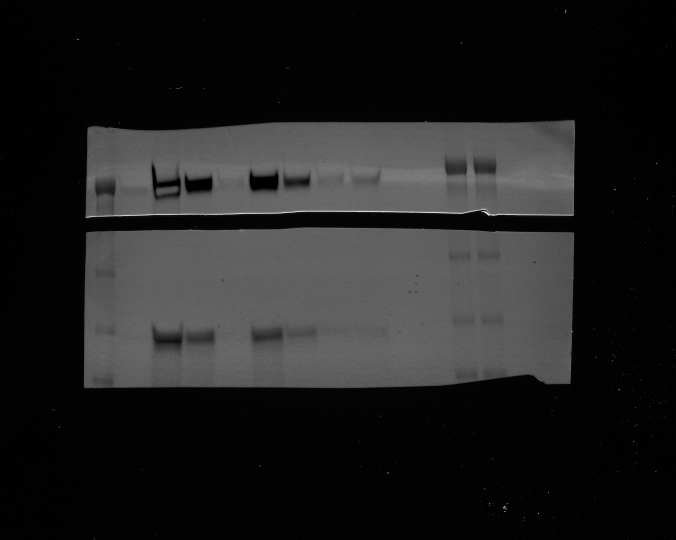

Supplement: Figure 3—figure supplement 1—source data 1. [file elife-80053-fig3-figsupp1-data1.zip › Figure 3 - Supplement 1- Source Data 1/Figure3-Suppl-B/Raw blots/Figure3-Suppl1-B_GCP5andgTUB_sourceOfgTuBband_800msec_MIDDLE.tif]

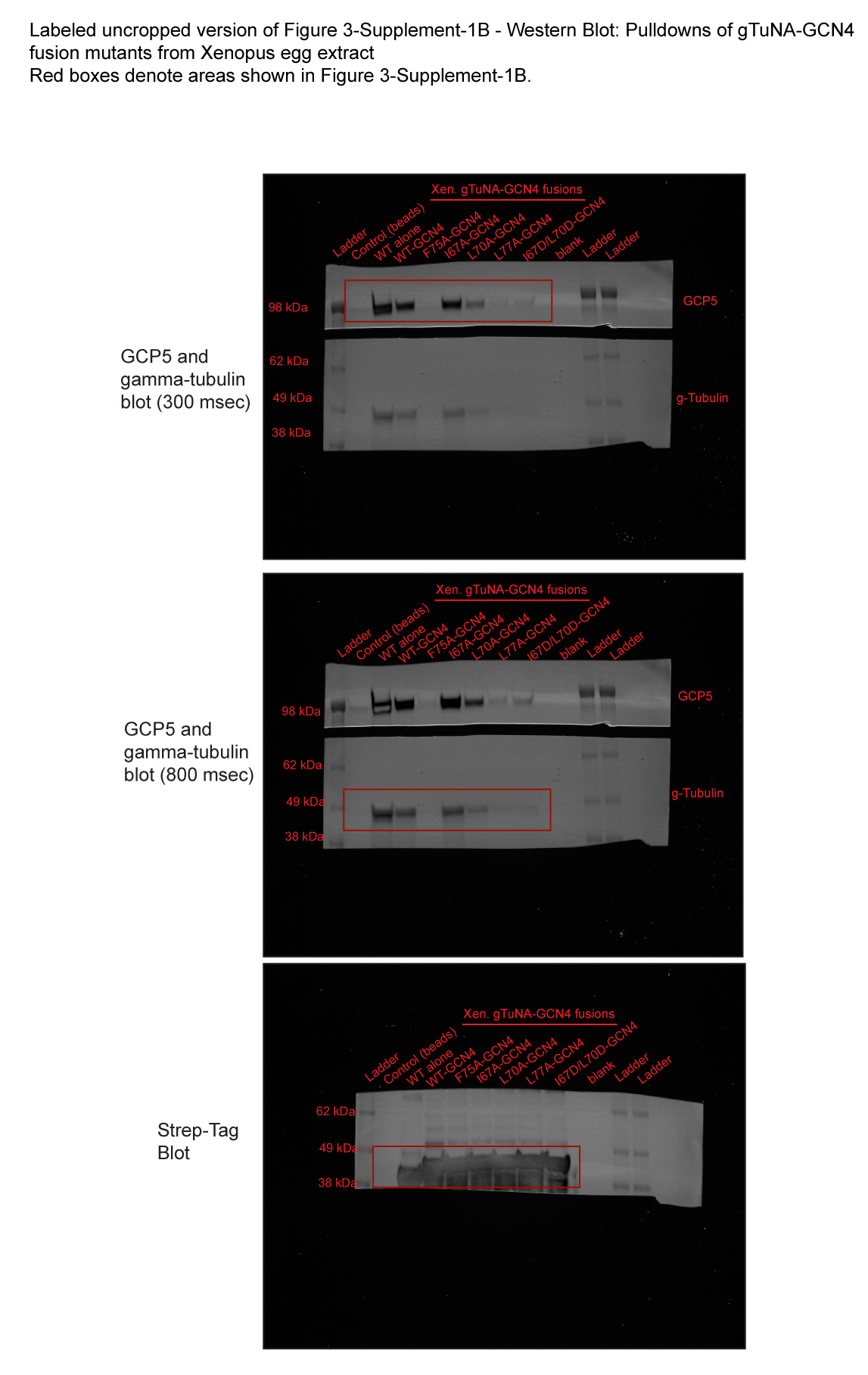

Supplement: Figure 3—figure supplement 1—source data 1. [file elife-80053-fig3-figsupp1-data1.zip › Figure 3 - Supplement 1- Source Data 1/Figure3-Suppl-B/Labeled_uncropped_Fig3-Suppl1-B-01.tif]
